# Privacy-Preserving Head Pose Estimation System for Measuring Cervical Range of Motion

**DOI:** 10.3390/s26165310

**Published:** 2026-08-21

**Authors:** Zhuofu Liu, Lichao Zhang, Gaohan Li, Peter W. McCarthy

**Affiliations:** 1 School of Measurement Control Technology and Communication Engineering, Harbin University of Science and Technology, Harbin 150080, China; 2 Faculty of Life Science and Education, University of South Wales, Treforest, Pontypridd CF37 1DL, UK; peter.mccarthy@southwales.ac.uk; 3 Faculty of Health Sciences, Durban University of Technology, Durban 1334, South Africa

**Keywords:** cervical range of motion, head pose estimation, deep learning, non-contact measurement, privacy preservation

## Abstract

Cervical range of motion (CROM) has been used in research and clinically for assessing cervical health. Gold-standard goniometers tend to be cumbersome. However, Inertial Measurement Units (IMUs) or vision-based alternatives demand frequent calibration and/or costly hardware; moreover, the subject is aware of being measured and there is a risk of breaching privacy. In response, we have developed a non-contact HPNet system for head pose estimation (HPE) that can use a rear-facing camera to quantify CROM accurately. A Re-parameterized Visual Geometry Group (RepVGG)-D2se model is employed as the backbone of the network, and a Spatial Feature Enhancement (SCFE) module is incorporated to improve feature extraction. HPNet was evaluated on the large-scale Carnegie Mellon University (CMU) Panoptic dataset, achieving a mean absolute error (MAE) of 3.48°, 3.22°and 3.34° for yaw, pitch and roll respectively. Inter-instrument reliability was excellent for all six cervical movements when compared with the research/clinical-grade CROM device, with intraclass correlation coefficients (ICCs) averaging 0.939. Bland–Altman plots confirmed close agreement between the two methods. Cervical movement trajectory curves further confirmed the concordance between the clinical device and our method. The system is fully automatic, requires only a rear-facing camera, effectively preserves patient privacy, and provides accurate cervical posture estimation. This technology may provide a basis for future applications in neck-disorder screening, remote health monitoring, and personalized musculoskeletal wellness management, although further task-specific clinical validation will be required. To date, HPNet has been validated primarily on a computer-based platform and has not yet been deployed on smartphones. Future work will focus on model lightweighting, mobile deployment, and cross-device adaptation to facilitate its practical implementation on mobile devices.

## 1. Introduction

Head pose estimation (HPE) is a pivotal research area in computer vision with wide-ranging applications across various domains of artificial intelligence, including human–computer interaction, facial recognition, driver monitoring, and Virtual/Augmented Reality [1]. Over the past decade, the emergence of deep learning has significantly advanced the accuracy of HPE, particularly in estimating full-range head orientations under unconstrained conditions [2]. These advancements have intensified research interest, positioning HPE as both a challenging and rapidly evolving field of research. The primary objective of HPE is to determine the 3D orientation of the human head, typically represented by three angular components: pitch, yaw, and roll [3]. Current research efforts are largely directed toward improving estimation accuracy, robustness, and real-time performance, especially in complex and dynamic environments. 

In recent years, the application of HPE has expanded into the clinical domain, particularly in the assessment and diagnosis of cervical spine disorders [2]. However, cervical range of motion (CROM) measurement still relies on manually operated devices that are examiner-dependent and prone to parallax error [4]. Wearable Inertial Measurement Unit (IMU)-based systems mitigate subjectivity, but introduce drift during prolonged recordings, demand precise sensor placement and affect user comfort [4,5]. Head-mounted devices such as Virtual Reality (VR) and Augmented Reality (AR) systems have also been explored for cervical mobility tracking [6,7]; however, such systems remain too intrusive and may raise privacy concerns. In all of these methods, the person being measured is aware of, if not directly affected by, the measurement. Consequently, there is a growing demand for objective (not dependent on a trained observer), non-invasive (not requiring attachment to the subject), marker-free measurement techniques, making vision-based HPE an increasingly attractive alternative.

By analyzing images or video sequences, HPE can indirectly infer cervical spine mobility through the estimation of head orientation angles [2,8]. Recent advances in deep learning, including Convolutional Neural Networks (CNNs) and Recurrent Neural Networks (RNNs), have provided robust tools for enhancing the precision and reliability of HPE in CROM assessments [9]. Furthermore, transformer-based architectures have been employed to improve estimation accuracy by capturing long-range dependencies in spatial and temporal data [10]. Despite these advancements, image-based HPE still faces significant practical challenges, such as issues generated by variations in lighting, partial occlusion of part of the image, and inter-individual anatomical differences. Researchers have proposed various strategies to mitigate these issues, including multi-camera setups to capture head pose from a number of viewpoints, thereby reducing the impact of occlusions and improving estimation robustness [11]. Meanwhile, data-augmentation strategies ranging from photometric perturbations to synthetically generated head trajectories are routinely exploited to enlarge the training corpus and, consequently, improve the capability of the model to be used more widely across different demographics and acquisition conditions [12].

The limitations of existing methods for CROM measurement include intra- and inter-rater variability, sensor drift in IMU-based systems, the invasiveness of traditional CROM devices (which need to be securely placed on the head), and privacy concerns caused by facial exposure in image-based HPE approaches. To overcome these, this study proposes a novel deep learning-based CROM measurement framework. The main innovations and practical significance of this study are summarized as follows:

(1) Accuracy—An advanced deep learning framework is employed to perform precise HPE from a single RGB image, enabling robust extraction of head pose features and significantly improving measurement accuracy.
(2) Privacy protection—A unique rear-facing image acquisition system is introduced to capture cervical pose from the occipital region, thereby obviating the need for facial capture and significantly reducing the risk of facial biometric data leakage.
(3) Generalization—The model is trained on the large-scale CMU Panoptic dataset [13], which provides diverse human posture and motion data. This enhances the robustness and generalizability of the proposed approach under complex neck movements.

The metrological fidelity and readiness were rigorously benchmarked against a research/clinical-grade CROM device [14,15,16]. The successful implementation of this study will bring multiple positive impacts and far-reaching implications to the field of cervical spine motion assessment. Firstly, our proposed low-cost, non-invasive measurement technique significantly lowers the economic burden of CROM measurement, thereby promoting its application in primary care, non-contact research and home settings. Secondly, both the lack of attachment of a physical device and introduction of a privacy protection mechanism are expected to enhance patient acceptance and compliance with CROM measurements, facilitating the widespread collection and analysis of cervical spine motion data. Such a dataset can contribute to a more comprehensive understanding of the epidemiological characteristics of cervical spine disorders, the patterns of disease progression, the impact of daily activity and the long-term effects of different treatment options [17,18]: for example, early screening for cervical spondylosis may be achieved through continuous monitoring of CROM changes, enabling timely identification of potentially at-risk individuals and guiding early intervention [19]. Arguably, a more important application of such a system lies in the study and diagnosis of disorders affecting head movement, such as tremor and dystonia, as well as in assessing the severity of head impacts in sports [20]. The method may also provide a quantitative basis for the objective assessment of the effectiveness of rehabilitation therapy, enabling clinicians to adjust the treatment plan more accurately and optimize the recovery process of patients. In addition, this study designed and validated a privacy-preserving head pose estimation model based on rear-view images of the human. The model holds promise for applications in motor system neurology to characterize complex head-motion patterns in disorders including cervical dystonia [20], though further validation with dynamic continuous tracking and quantitative trajectory metrics across all subjects is warranted. Finally, this study explores the cross-application of deep learning in healthcare, which is expected to promote the further development of AI technology in clinical diagnosis and monitoring, and lay the foundation for building a more intelligent healthcare management system [21].

## 2. Related Work

HPE quantifies the three rotational degrees of freedom—yaw, pitch, and roll—that completely specify the orientation of the head. Below we survey the literature with emphasis on these angular variables, their operational ranges, sensing modalities, and the deep-learning algorithms now dominant in the field [22]. Existing methods can be clustered into two paradigms: landmark-based [23,24] and landmark-free [22,25,26,27]. The former locates fiducial points on the face and then computes pose via geometric constraints, delivering high accuracy when landmarks are visible but degrading sharply under occlusion. Landmark-free approaches, in contrast, infer pose directly from image features, trading a modest loss in precision for substantially greater robustness.

### 2.1. Head Pose Representation

**Figure 1:**
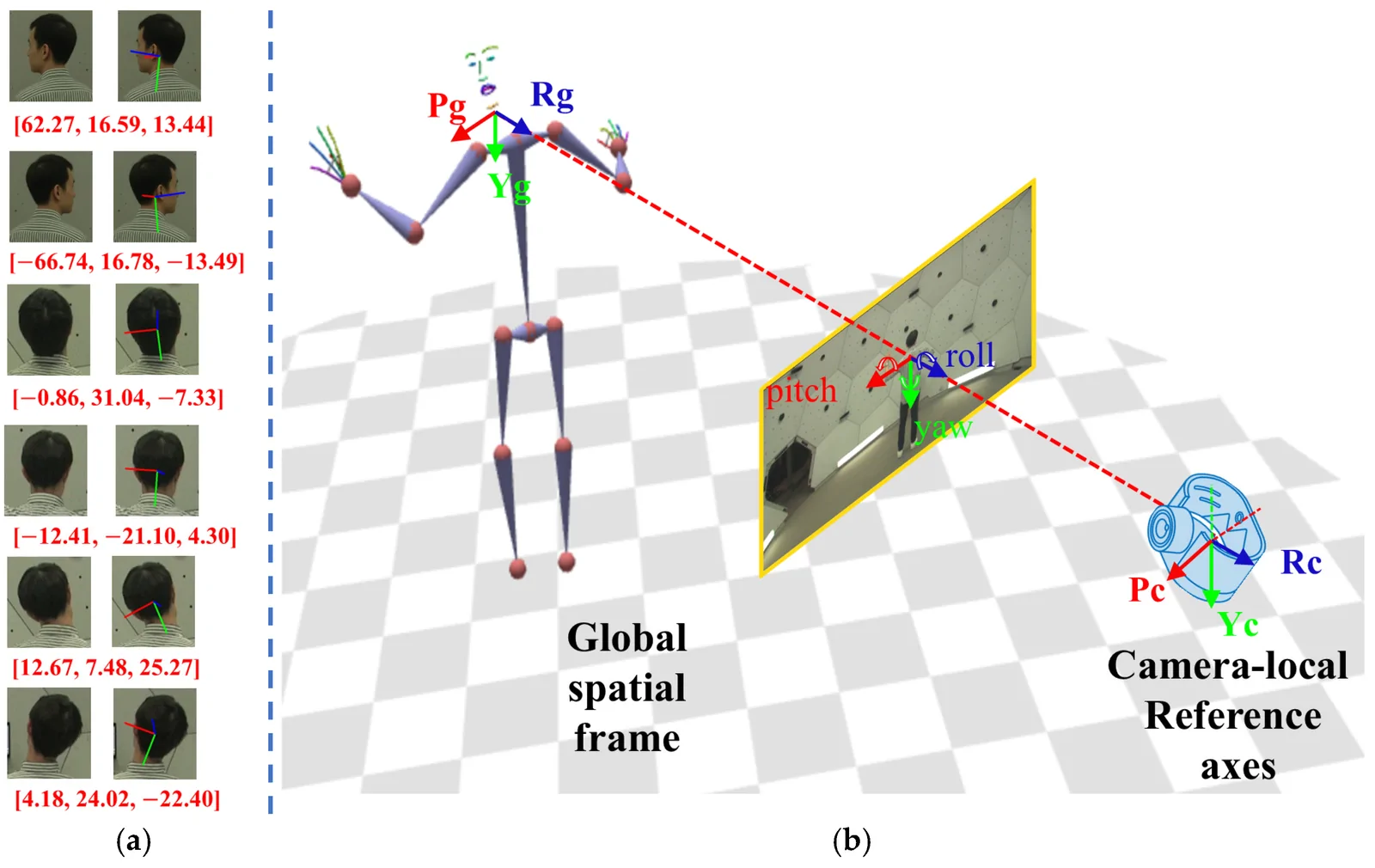
Coordinate system of HPE. (**a**) Examples of estimated head poses with corresponding pitch, yaw, and roll angles; (**b**) global spatial coordinate system and camera-local reference axes used to define head pose angles.

A critical design choice in HPE is the mathematical encoding of rotation. Euler angles, which decompose the orientation into successive pitch, yaw, and roll rotations relevant to the camera axes (Figure 1), remain the default choice because of their apparent simplicity. Yet their spherical topology conceals a fundamental flaw: gimbal lock, a singularity that collapses two rotational axes into one and destabilizes gradient-based learning [28]. Quaternions eliminate this singularity but inherit antipodal ambiguity; in wide-range head pose settings this symmetry can fragment the loss surface and degrade convergence [29].

To overcome this challenge, Beyer et al. [30] encoded yaw as a biternion (the unit vector of the 2D rotation matrix), achieving periodic smoothness in a single angular dimension. This idea was extended into 3D space by representing orientation with the first two columns of the rotation matrix [31]. The representation has been utilized in both face reconstruction [26] and head alignment [32].

### 2.2. Landmark-Based Approaches

Most landmark-based approaches estimate head pose using the facial features as the landmarks [22,23,24,25,26]; for instance, Kao et al. [26] employed a Perspective-n-Point method to regress head poses from landmarks, while Hsu and Chung [27] localized eye centers using geometric transformations and image translation learning. The HyperFace model [28] integrates four tasks, namely face detection, landmark localization, pose estimation, and gender recognition, into a single unified framework.

Additionally, Yu et al. [29] proposed a tracking initialization method via facial detection and landmark localization, while Bisogni et al. [33] developed the FrActal-Based Strategy for Head pose Estimation (FASHE) model to enhance facial landmark prediction. Based on ResNet-50 and multi-task cascaded convolutional networks, the end-to-end Lie Algebra Residual Network (LARNeXt) model for face recognition [30] implemented landmark detection and facial recognition simultaneously. Similarly, Barra et al. [22] proposed a two-stage cascaded approach: it first predicts 68 facial landmarks, then assigns these landmarks to specific facial sectors using a web-shaped model to boost HPE accuracy. 

While these methods achieve high accuracy for small angular movements (less than ±30°) [30,31], they rely heavily on precise landmark detection. This dependency makes them vulnerable to inaccuracies when facial landmarks are occluded or under extreme head rotations. As a result, their effectiveness is limited for full-range HPE tasks that involve large-angle rotations. To address these drawbacks, recent studies have shifted focus to landmark-free deep learning models, which estimate head pose directly from image features to improve robustness.

### 2.3. Landmark-Free Approaches

Landmark-free head pose estimators discard facial key points entirely and infer orientation directly from the full image [32,33,34,35,36,37]. To estimate head pose from a single image, Yang et al. [32] developed a fine-grained structure with the help of soft stagewise regression and by spatially grouping features before aggregation. The fine-grained structure provides part-based information and pooled values. By utilizing learnable and non-learnable importance over the spatial location, different model variants can be generated and form a complementary ensemble. Liu et al. [34] counter low pose tolerance and representation ambiguity with a three-branch architecture that couples a triplet module with a matrix Fisher distribution module. The triplet module pulls embeddings of identical poses together while pushing embeddings of different poses apart. Concurrently, the matrix Fisher module treats head rotation as a random variable, whose uncertainty is captured by an exponential probability density.

To mitigate perspective distortion caused by the imperfect alignment between the people and the camera, an image-rectification method [36] is used to compute the angle between the optical axis of the camera and the projection vector of the center, then undistorts the view with a perspective transform. For occluded scenarios, Celestino et al. [37] proposed a CNN-based latent-space regression framework. During training, each head image is presented with a synthetically occluded version and any resultant consistency loss compels the network to produce nearly identical latent codes for the two versions, forcing it to stay robust when much of the object is hidden. However, most models still parameterize rotation with Euler angles and consequently suffer gimbal lock, leading to the loss of a degree of freedom in extreme orientations.

To address this, recent works have adopted alternative representations. For instance, 6DHPENet [35] employs 6D orthogonal components to avoid singularities, while MFDNet [34] and TriNet [38] regress three mutually orthogonal unit vectors and reconstruct the matrix through the Gram-Schmidt process. To eliminate the ambiguities of discrete rotation labels, Hempel et al. [33] adopted a rotation matrix formalism that allows the network to regress rotation directly. A geodesic-distance loss, aligned with the manifold geometry, further penalizes deviations and yields efficient and robust estimates.

## 3. Methods

Initially, we introduced the HPNet model to predict the rotation matrix from which pitch, yaw, and roll angles are derived. Then, the network was trained and validated on the CMU Panoptic dataset and subsequently employed for CROM (Performance Attainment Associates, Lindstrom, MN, USA) assessment. Finally, the accuracy, variability and reliability of the HPNet-based CROM measurements were statistically evaluated against a research/clinical-grade reference device.

### 3.1. HPNet

**Figure 2:**
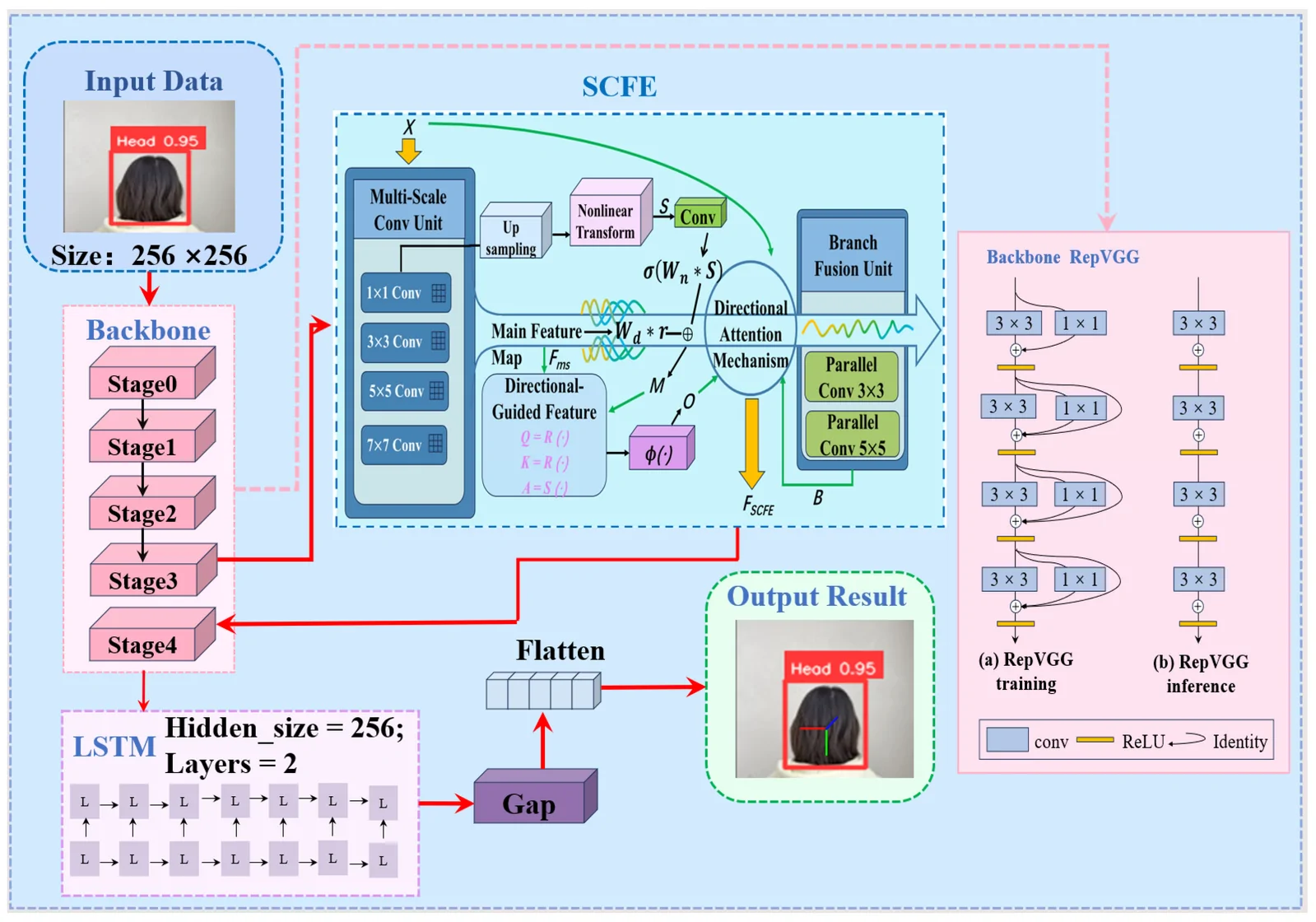
Architecture of the proposed HPNet model including the backbone, Spatial Channel Feature Extraction (SCFE) module and Long Short-Term Memory (LSTM) module. RepVGG: Re-parameterized Visual Geometry Group; ReLU: Rectified Linear Unit.

The proposed HPNet model (Figure 2) integrates three core components: (1) a Re-parameterized Visual Geometry Group (RepVGG) backbone [39] that leverages re-parameterization to achieve high training accuracy, (2) a Spatial Channel Feature Extraction (SCFE) module that enhances spatial awareness and motion perception in 6D rotation estimation, and (3) a Long Short-Term Memory (LSTM) module for 6D feature regression. In the current implementation, the LSTM processes each frame independently with a sequence length of 1, functioning not as a temporal model but as a gated feature refinement unit. Its forget, input, and output gates adaptively filter and modulate the high-dimensional feature vector before the final fully connected layer, providing implicit regularization and suppressing pose-irrelevant noise [40]. Compared with a simple fully connected regression layer, this gated structure provides an adaptive feature-selection mechanism rather than applying a fixed linear transformation to all feature channels. This is particularly useful for rear-view head images, where pose-related cues may be subtle and easily affected by hair, head shape, and background variation. The ablation results further support this design, as incorporating the LSTM module consistently reduced the MAE values for yaw, pitch, and roll. In the proposed YOLO26–HPNet pipeline, YOLO26 [41] is used as the front-end head localization module to detect the occipital region from full-frame rear-view images and provide region-of-interest inputs for HPNet. During the development of the detection module, multiple object detection frameworks, including YOLOv8, YOLOv10, YOLO26, and RT-DETR, were investigated as candidate solutions for head region localization. Based on the comparative analysis of these models and the requirements of the current task, YOLO26 was ultimately selected as the front-end detection framework. HPNet then serves as the core pose estimation module to regress yaw, pitch, and roll angles from the localized occipital region. In the controlled validation experiments, the camera-to-subject distance was fixed and the head region was consistently visible; therefore, the validation mainly focused on the HPNet-based pose estimation performance, while YOLO26 was used as the front-end localization component of the complete system pipeline.

The SCFE module introduces a novel design, operating on intermediate RepVGG feature maps through a multi-stage process. The process is as follows: First is a multi-scale convolutional unit that aggregates spatial information across diverse receptive fields (1 × 1, 3 × 3, 5 × 5, 7 × 7), generating a comprehensive representation of both local details and global context. This is followed by a context generator and nonlinear transformation layer, which produces an intermediate spatial map that is further refined via dilated convolution to yield directional guidance. This directional guidance interacts with the primary feature map through a directional attention mechanism, enabling direction-sensitive feature enhancement. Finally, a branch fusion unit supplements the representation with parallel convolutional branches and re-injects the enhanced features into the main backbone. This architecture empowers SCFE to capture both static spatial dependencies and motion cues, thereby substantially improving the robustness of rotation estimation.

To better illustrate the internal operations of the SCFE module, the following mathematical formulations describe its key components in detail, including the multi-scale convolutional aggregation, normalization, attention and fusion mechanisms. The following formulations clarify how spatial and motion cues are jointly encoded to enhance the feature representation.

(1) Multi-Scale Feature Fusion

For the input feature map $X\in R^{N\times C\times H\times W}$, the batch size is N, the number of channels is C, and the spatial dimensions are H and W. Four different convolutional kernels are used to extract distinct information:

(1) $$F_{i}=B\left(W_{i}*X\right)\;i\in \left\{1,3,5,7\right\}$$

(2) $$F_{ms}=\emptyset \left(Concat\left[F_{1},F_{3},F_{5},F_{7}\right]\right)$$

where X is the input feature map from Stage 3; $W_{i}$ represents the convolutional kernel with a size of i×i; B(·) is the BN (Batch Normalization) + ReLU (Rectified Linear Unit) operation; ∗ refers to the convolution operation; Concat[·] signifies channel concatenation; and ∅(·) represents the 1×1 convolution + BN + ReLU fused module.

The convolution operation (∗) is calculated using the following formula:

(3) $${\left(W\;*\;X\right)}_{c,i,j}\;=\;\sum_{m=1}^{C_{in}}\;\sum_{u=-\left|\frac{k_{h}}{2}\right|}^{\left|\frac{k_{h}}{2}\right|}\;\sum_{v=-\left|\frac{k_{w}}{2}\right|}^{\left|\frac{k_{w}}{2}\right|}W_{c,m,u,v}X_{m,i+u,j+v}$$

where $k_{h}$ and $k_{w}$ denote the height and width of the convolutional kernel, while i and j denote the indices of the input feature map. The calculation formula for BN is given by:

(4) $$BN\left(x_{n,c}\right)=\gamma_{c}\frac{x_{n,c}-\mu_{c}}{\sqrt{\sigma_{c}^{2}+\varepsilon }}+\beta_{c}$$

where *σ*(·) denotes a nonlinear activation function consisting of Batch Normalization (BN) followed by ReLU; $\mu_{c}$ denotes the mean of the mini-batch; $\sigma_{c}^{2}$ represents the variance of the mini-batch; $\gamma_{c}$ and $\beta_{c}$ are the learnable scaling and translation parameters; ε is a small constant to prevent division by zero; and $x_{n,c}$ denotes the set of all spatial positions (i,j) in batch n and channel c.

(2) Context Generator

Multi-scale features undergo upsampling and channel compression to obtain spatial context maps:

(5) $$S=\sigma \left(W_{s}*Up\left(F_{ms}\right)\right)$$

where $Up\left(\cdot \right)$ denotes bilinear upscaling; $W_{s}$ represents 3×3 convolution; and $\sigma \left(\cdot \right)$ signifies the BN + ReLU nonlinear activation. S represents spatial relationships, serving as the foundation for directional guidance.

(3) Directional-guided Feature

The directional-guided feature is obtained through two independent convolutional transformations which are performed on the input feature $r\in R^{C\times H\times W}$ and the spatial context S, respectively:

(6) $$M=W_{d}*r+\sigma \left(W_{n}*S\right)$$

where $W_{d}$ denotes a 3×3 convolution with a dilation rate of 2, $W_{n}$ denotes a 1×1 convolution, σ(⋅) signifies the nonlinear activation and M represents the directional-guided feature.

(4) Directional Attention

The core cross-domain attention mechanism computes weights using the directional- guided feature M and the backbone features $F_{ms}$:

(7) $$\begin{array}{c} Q=Reshape(M)\in R^{N\times D\times H\times W},\\ K=Reshape(\emptyset (F_{ms}))\in R^{N\times C\times H\times W},\\ A=Softmax(\max\left(KQ^{T}\right)-KQ^{T}) \end{array}$$

where A is the direction-aware attention matrix; N is the batch size; C denotes the number of channels; D represents the number of directional-guided channels; and H and W denote the spatial dimensions. The Reshape operation does not alter element values but remaps tensor elements into a new dimensional structure, typically via the row-major or column-major order. This operation satisfies the identity $Y_{j_{1},\ldots \ldots ,j_{m}}=X_{i_{1},\ldots \ldots i_{k}}$. First, the multi-dimensional indices $\left(i_{1},\ldots \ldots ,i_{k}\right)$ of X are converted into a one-dimensional index p as follows:

(8) $$p=1+\sum_{r=1}^{k}\left(i_{r}-1\right)\prod_{s=r+1}^{k}d_{s}$$

where k denotes the number of dimensions of the input tensor X, $i_{r}$ denotes the 1-based index of X along its r-th dimension, $d_{s}$ denotes the size of the s-th dimension of X, and p denotes the 1-based linear position index of the accessed element in the flattened input tensor, corresponding to its linear offset.

This one-dimensional index p is then mapped to multi-dimensional indices $\left(j_{1},\ldots \ldots ,j_{m}\right)$ of the output tensor Y as follows:

(9) $$j_{t}=1+\left\lfloor \frac{\left(p-1\right)\;\mathrm{mod}\;\left(\prod_{s=t}^{m}e_{s}\right)}{\prod_{s=t+1}^{m}e_{s}}\right\rfloor ,\;t=1,\ldots ,m.$$

where $e_{s}$ denotes the size of the s-th dimension of the output tensor *Y*. Then, the directional-guided feature M is used to perform the weighted sum operation via the attention matrix *A*:

(10) $$O=\varphi \left(A\cdot \mathrm{Reshape}\left(M\right)\right)$$

where Reshape(·) rearranges the directional-guided feature *M* into a matrix representation suitable for the attention operation while preserving the feature values. The multiplication *A*·Reshape(*M*) applies the direction-aware attention weights to the feature representation. The function *φ*(·) denotes the output projection operation, which maps the attention-enhanced feature back to the main branch dimension.

### 3.2. Rotation Matrix

To estimate the angles of the 3D head rotations, the first step is to choose the rotation representation. The rotation matrix R is a 3 × 3 matrix projected onto the special orthogonal group SO(3) with the orthogonality constraint ${RR}^{T}=I$, where $R^{T}$ is the transpose of matrix R and I is the identity matrix. Directly regressing all nine parameters of R can be computationally intensive and may lead to the gimbal-lock phenomenon [33,42].

To address the above difficulties, this study employed the 6D rotation representation proposed by Zhou et al. [42] and subsequently adopted for head pose estimation by Hempel et al. [33]. Zhou et al. [42] theoretically showed that 3D rotations can be continuously represented in 5D and 6D spaces, whereas conventional representations such as Euler angles and quaternions may suffer from discontinuity in neural-network-based regression. In this study, the 6D representation was adopted because it provides a more direct and practical reconstruction process than the 5D representation. Specifically, although the 5D representation is more compact, it generally requires an additional stereographic unprojection step before reconstructing the rotation matrix [42]. By contrast, the 6D representation represents rotation using two unconstrained 3D vectors, which can be directly converted into a valid rotation matrix through normalization, orthogonalization, and cross-product operations, as formulated in Equation (12). This simple and continuous reconstruction process helps avoid invalid rotation outputs and provides more stable and accurate rotation regression for wide-range rear-view head pose estimation. In lieu of conventional three-dimensional Euler angles, the 6D rotation representation encodes orientation as a pair of unconstrained 3D vectors, $\alpha_{1}\;and\;\alpha_{2}$. As shown in Figure 2, the proposed HPNet outputs a 6D rotation representation (α_1_, α_2_), which is then converted into a valid rotation matrix R via the Gram–Schmidt process described in this section. These two vectors are then orthonormalized via the Gram–Schmidt process to form the first two rows of the rotation matrix, while the third row is obtained by their cross product, ensuring a valid 3×3 rotation matrix:

(11) $$R={\left[\begin{array}{ccc} - & \beta_{1} & -\\ - & \beta_{2} & -\\ - & \beta_{3} & - \end{array}\right]}^{T}$$

where $\beta_{1},\beta_{2},$ and $\beta_{3}$ represent the three orthogonal basis vectors of the rotation matrix, which are derived by orthogonalizing $\alpha_{1}$ and $\alpha_{2}$.

(12) $$\begin{array}{c} \beta_{1}=\frac{\alpha_{1}}{\left\|\alpha_{1}\right\|}\\ u_{2}=\alpha_{2}-(\beta_{1}\cdot \alpha_{2})\beta_{1}\\ \beta_{2}=\frac{u_{2}}{\left\|u_{2}\right\|}\\ \beta_{3}=\beta_{1}\times \beta_{2} \end{array}$$

where $\alpha_{1},\;\alpha_{2}$ are directly predicted by the HPNet, $\left\|\alpha_{1}\right\|$ denotes the Euclidean norm of $\alpha_{1}$, $\beta_{1}\cdot \alpha_{2}$ represents the inner product between $\beta_{1}$ and $\alpha_{2}$, and $\beta_{1}\times \beta_{2}$ denotes the cross product used to obtain $\beta_{3}$. Equation (12) shows that the 6D output can be mapped to SO(3) through only normalization, orthogonalization, and cross-product operations. This direct reconstruction process is the main reason why the 6D representation is adopted in HPNet instead of the more compact but less direct 5D representation. The difference between the ground truth and predicted values during training is calculated by the geodesic distance loss function [33]:

(13) $$L_{GD}={cos}^{-1}\left(\frac{tr\left(\varphi_{p}\varphi_{gt}^{T}\right)-1}{2}\right)$$

where $\varphi_{p}$ denotes the rotation matrix predicted by the network, $\varphi_{gt}$ represents the ground-truth rotation matrix, $\varphi_{p}$ and $\varphi_{gt}\in$ SO(3), and $tr\left(\cdot \right)$ denotes the trace of a matrix.

### 3.3. Training Dataset

We used the CMU Panoptic dataset [43], which contains fully rotated head images with corresponding annotations. In this dataset, participants perform arbitrary tasks inside a geodesic dome equipped with 31 evenly spaced HD cameras. While the dataset primarily targets human pose capture, it also provides 3D facial landmark annotations, enabling head pose (position and relative orientation) labels to be extracted from multiple camera viewpoints [44]. First, we extracted the coordinates of each head bounding box and corresponding Euler angle labels (pitch, yaw, roll). Subsequently, based on the labeled bounding box information, we selected the head region from the original high-resolution images and uniformly resized all the head images to 256 × 256 pixels. This preprocessing ensures the normalization of the training data while preserving the complete head pose information. The dataset was partitioned into training and validation sets to prevent data leakage. Eventually, we obtained 31,543 training samples and 9681 validation samples from the CMU Panoptic dataset. The above data partitioning was naturally determined by subject-level splitting, ensuring that the two subsets are mutually independent. Furthermore, since the pose annotations of the CMU Panoptic dataset are derived from multi-view 3D reconstruction and defined in a unified global coordinate system, the labels are independent of any single camera viewpoint and remain accurate and consistent even under rear-view conditions.

### 3.4. Experimental System for CROM Measurement

**Figure 3:**
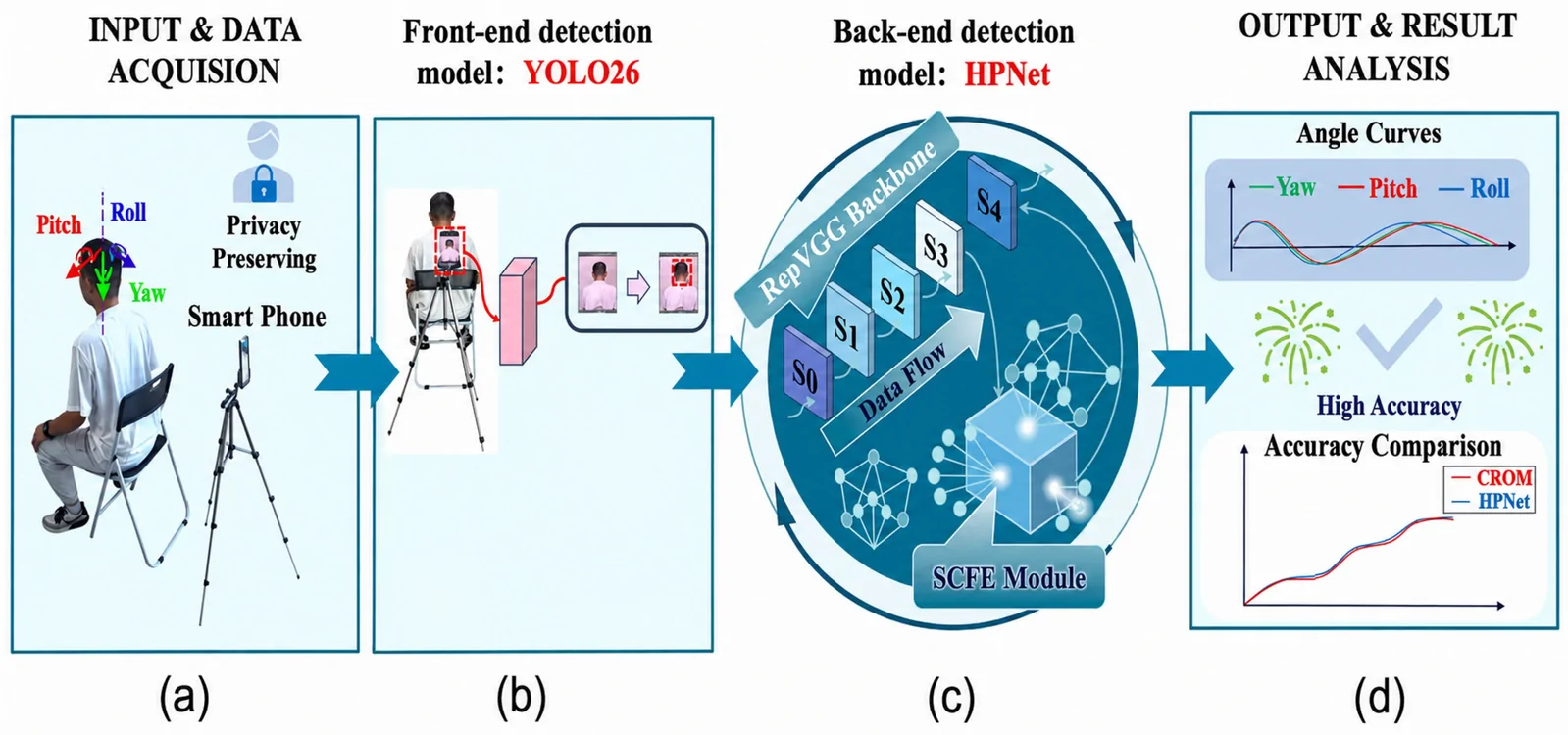
Experimental configuration: (**a**) CROM acquisition system: the participant is fitted with a research/clinical-grade CROM instrument supplemented by a pair of strong magnets to create a magnetic field that was used to align the upper goniometer (which is a compass needle) and to prevent interference with the readings from the earth’s magnetic field as well as negate any effects of shoulder movement (so that only movement resulting from the neck was being measured). (**b**) Rear-view (occipital) image acquisition and region-of-interest preparation; YOLO26 can be used for automatic head localization in practical deployment. (**c**) High-precision head pose estimation backbone network based on RepVGG, incorporating hierarchical feature flow (S0–S4) and SCFE module for refined feature enhancement and pose regression. (**d**) Visualization of pose estimation results and performance validation, including accuracy comparison between the proposed HPNet and the CROM device.

As depicted in Figure 3a, participants were instructed to wear the research/clinical-grade CROM device [45] and maintain a standardized seated posture: upright on an armless chair, hands resting on knees, gaze forward. The CROM device served as the reference standard. To capture cervical movements, a tripod-mounted Android smartphone was positioned 60 cm behind the participant, with its optical axis orthogonal to the coronal plane (Figure 3b). The tripod height was adjusted to ensure the entire occiput remained within the camera’s field of view. The smartphone camera resolution was set to 1920 × 1080 pixels.

### 3.5. Experimental Procedures for CROM Measurement

In this study, no explicit coordinate transformation was applied. Instead, the alignment between the CROM device and the camera coordinate system was achieved through a unified reference posture. Specifically, at the beginning of each trial, all participants were instructed to maintain a standard neutral head position, at which point both the HPNet output and the CROM device measurements were set to zero (i.e., the neutral position was defined as a common 0° reference for both systems). All subsequent head motion angles were then measured relative to this neutral position and expressed as angular increments, thereby eliminating differences in absolute coordinate systems between the two measurement methods under a unified reference framework.

Two complementary experiments were designed to validate the HPNet system. Both experiments were approved by the Faculty Research Committee (No. 20241118) and conducted in accordance with the guidelines outlined in the Declaration of Helsinki. All participants provided informed consent after receiving a verbal and written description of the experimental procedures. Experiments were conducted from 7 June to 22 August 2025.

Participants in this study were healthy adults aged 18–27 years who had no history of previous treatment for neck pain and had been pain-free for at least one year [46]. Individuals who had difficulty maintaining or transitioning between specific cervical postures were also excluded. Before the trials, all participants were required not to engage in intense physical activity for 48 h.

**Experiment** **1** (**feasibility test**)**.** *A male volunteer (24 y, 173 cm, 80 kg) and a female volunteer (24 y, 175.5 cm, 45 kg) were randomly selected from the recruited participants to perform the six cervical movements described below:*

*Forward Flexion (FF) and Backward Extension (BE) in the sagittal plane, Left Lateral Flexion (LLF) and Right Lateral Flexion (RLF) in the coronal plane, and Left Rotation (LR) and Right Rotation (RR) in the transverse plane.*

For each movement, the participant wore the research/clinical-grade CROM device and sequentially tilted or rotated their head in 4-degree increments, starting from the initial position. Since the CROM device has a 2° resolution, the 4° step ensured measurable and consistent changes. At each target angle (e.g., 4°, 8°, 12°, etc.), the participant briefly held the position. Once the target angle was confirmed by the CROM device, the device was gently removed and then the rear camera took a photograph of the head posture. The interval from CROM device removal to photograph completion was controlled within approximately 5 s. To quantify the potential head posture change during this interval, a pre-experiment was conducted in which 3 participants performed 5 repeated device-removal–hold–photograph operations, yielding a mean posture drift of 1.2° (SD = 0.6°), which is below the 2° measurement resolution of the CROM device. To ensure reliability, this procedure was repeated three times, with adequate rest between trials. Before each measurement trial, participants maintained the neutral position, at which point both the HPNet output and the CROM device reading were set to zero reference. All subsequent angles were computed as increments relative to this neutral position, thereby eliminating differences in absolute coordinate systems between the two measurement methods. After image acquisition, HPNet was applied to estimate the neck angles [47]. These estimates will be compared with the corresponding CROM device readings.

**Experiment** **2** (**reliability test**)**.** A priori power analysis (G*Power 3.1.9.7) was conducted to ensure adequate sample size and statistical power. The power analysis parameters were set as follows: a single group, three repeated measures, a medium effect size (f = 0.25), a significance level of α = 0.05, a desired power of 0.80, and a correlation of 0.5 among repeated measures. The analysis indicated that at least 28 participants were required to achieve 80% statistical power. To ensure robustness, a total of 40 volunteers (20 males and 20 females) were recruited (Table 1*).*

**Figure 4:**
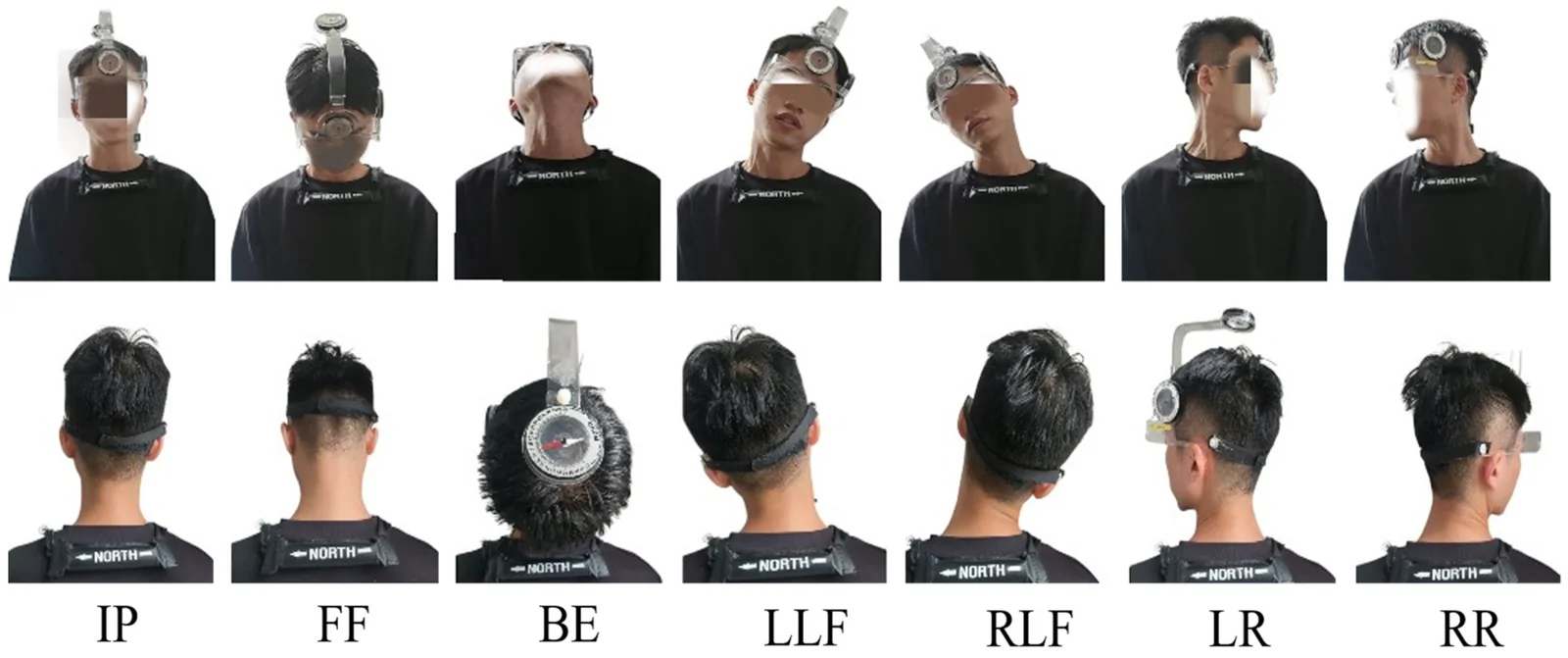
Six neck movements and initial states that volunteers need to perform. The specific pose abbreviations are: IP (initial or neutral position), FF (Forward Flexion), BE (Backward Extension), LLF (Left Lateral Flexion), RLF (Right Lateral Flexion), LR (Left Rotation), and RR (Right Rotation). The top row shows the frontal views of the cervical spine for each movement while the bottom row displays the rear views of the same movements.

During the experiment, participants were instructed to perform maximal head rotations in six directions: FF, BE, LLF, RLF, LR, RR (Figure 4). Each movement was repeated three times. Upon reaching the maximal angular displacement, participants maintained the position briefly while the CROM reading was recorded. The CROM device was then gently removed and the rear camera took a photograph of the head posture.

### 3.6. Statistical Methods

For the model training procedure, the mean absolute error (MAE) is used as the evaluation metric [48]:

(14) $$MAE=\frac{1}{n}\sum_{j=1}^{n}\left|\varphi_{j}-{\hat{\varphi }}_{j}\right|$$

where $\varphi_{j}$ is the ground truth angle and ${\hat{\varphi }}_{j}$ is the prediction angle. Furthermore, we evaluated the HPNet performance against the research/clinical-grade CROM device using root-mean-square error (RMSE):

(15) $$RMSE=\sqrt{\frac{1}{N}\sum_{i=1}^{N}{\left(y_{i}-{\hat{y}}_{i}\right)}^{2}}$$

where $y_{i}$ is the reference measurement by the CROM device; ${\hat{y}}_{i}$ is the HPNet prediction value; and N is the number of samples. The agreement between HPNet-derived angles and the CROM device is evaluated through Bland–Altman plots with 95% limits of agreement (LoAs) and intraclass correlation coefficients (ICCs) [49].

## 4. Results

The HPNet model was implemented using PyTorch 2.1.0 and trained on an NVIDIA RTX 4090 GPU. The network was trained using the Adam optimizer with an initial learning rate of 1 × 10^−4^ and a batch size of 20, for 100 epochs. These hyperparameters were selected based on the convergence behavior observed during training. During hyperparameter selection, several initial learning rates, including 1 × 10^−5^, 1 × 10^−4^, 5 × 10^−4^, and 1 × 10^−3^, were compared based on the training loss and MAE. A smaller learning rate of 1 × 10^−5^ resulted in slower convergence, whereas larger learning rates of 5 × 10^−4^ and 1 × 10^−3^ caused greater fluctuations in the training loss [50]. In comparison, an initial learning rate of 1 × 10^−4^ provided more stable optimization for 6D rotation regression and resulted in better overall performance. Therefore, it was adopted in this study. The total number of training epochs was set to 100 because model performance tended to stabilize before the end of training, with no obvious improvement observed in later epochs. A MultiStepLR scheduler was employed with milestones at epochs 10 and 20, reducing the learning rate by a factor of 0.5 to further refine the model after the initial convergence stage. Rear-view images from the CMU Panoptic dataset were used for model training and evaluation, and all input images were resized to 256 × 256 pixels. To further explore how batch size impacts HPNet’s prediction performance, we conducted experiments with batch sizes set to 80, 40, 20, 2, and 1. Model performance was assessed via per-axis MAE (yaw, pitch, roll) and average MAE; a batch size of 20 yielded the best overall performance.

### 4.1. Experimental Results for the CMU Panoptic Dataset

**Figure 5:**
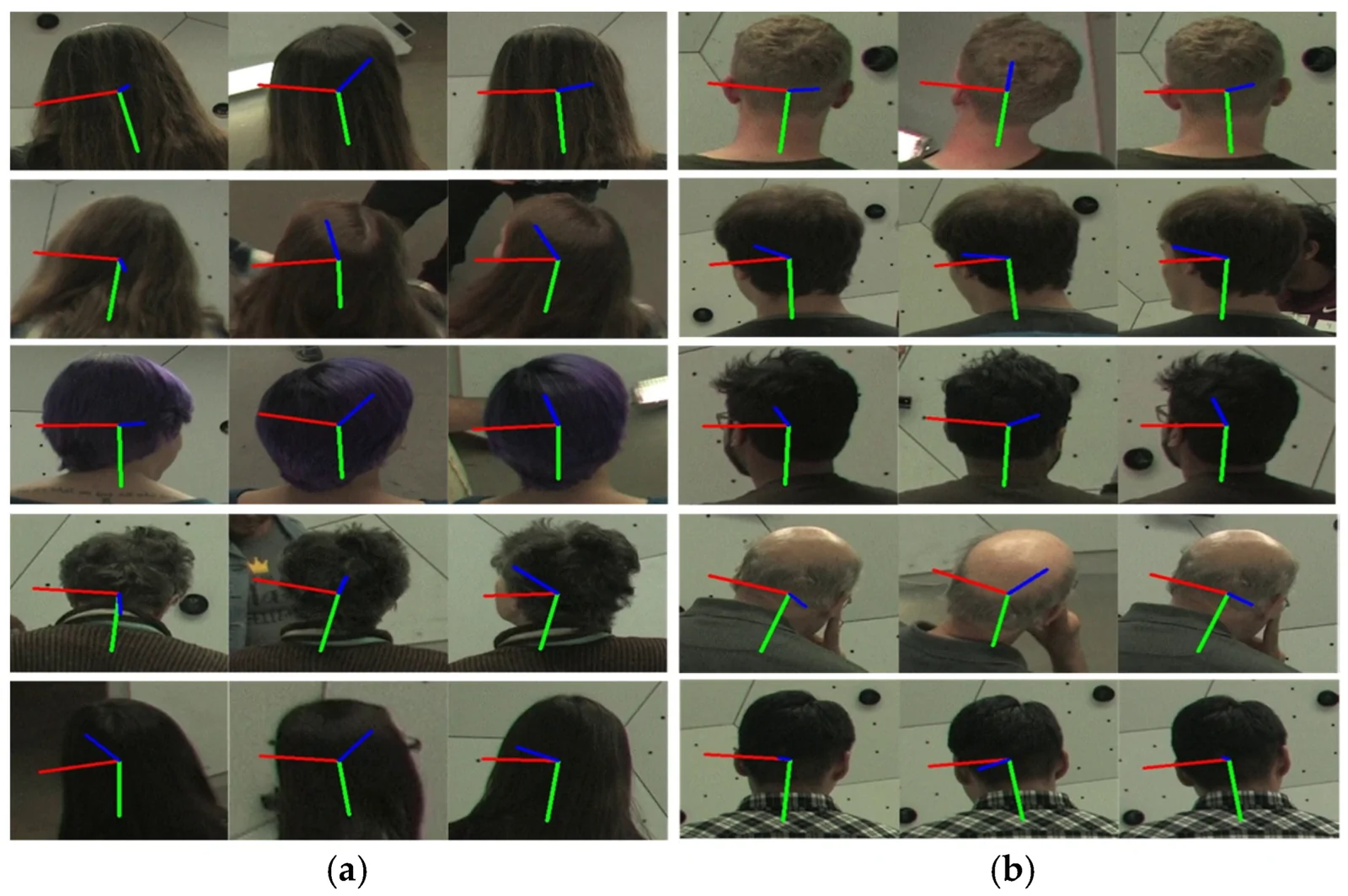
HPNet head movement tracking across age groups, split by gender: (**a**) female and (**b**) male subjects from the CMU Panoptic dataset.

Figure 5 illustrates the proposed HPNet’s ability in tracking head postures. The visualization uses green (yaw), red (pitch), and blue (roll) axes, consistent with the definition in Figure 1. To protect privacy, only rear-view images were employed for model training and validation. The results demonstrate that HPNet accurately tracks head movements, regardless of hair color or length.

To evaluate both estimation performance and model complexity, the head pose angle estimates of HPNet were compared with several state-of-the-art algorithms using MAE as the metric for yaw, pitch, and roll, respectively. In addition, Avg MAE was reported as the arithmetic mean of the yaw, pitch, and roll MAEs, providing an overall measure of angular estimation performance. The numbers of trainable parameters, reported in millions, were calculated based on the model implementations used in our experiments and are included in Table 2. Models marked “No” in the “Retrained” column indicate that the reported results were directly adopted from the original publication [39], where the models were trained and evaluated using frontal-view images from the public CMU Panoptic dataset. Models marked “Yes” were retrained and evaluated using our rear-view CMU Panoptic dataset split.

### 4.2. Ablation Study

We conducted comprehensive ablation experiments on the CMU Panoptic dataset to investigate the contributions of different components in HPNet (Table 3). Specifically, the experiments evaluated the effects of different backbone architectures and the incorporation of the SCFE and LSTM modules. All model variants were evaluated using the mean absolute errors (MAEs) for yaw, pitch, and roll. The average MAE (Avg MAE) was calculated as the arithmetic mean of the three angle-specific MAE values. 

Using RepVGG-B1g4 as the backbone, we quantitatively analyzed the individual contributions of the SCFE and LSTM modules, as well as their synergistic effect. On the CMU Panoptic dataset, compared with the baseline model without additional modules, incorporating SCFE alone reduced the MAE values of yaw, pitch, roll, and Avg MAE from 6.38°, 7.65°, 7.28°, and 7.10° to 6.25°, 7.56°, 7.22°, and 7.01°, respectively. Similarly, incorporating LSTM alone reduced these values to 6.19°, 7.58°, 7.17°, and 6.98°, respectively. When both SCFE and LSTM were integrated, the MAE values were further reduced to 5.86°, 7.21°, 6.57°, and 6.55°, corresponding to total reductions of 0.52°, 0.44°, 0.71°, and 0.55° compared with the baseline model. These results demonstrate that SCFE and LSTM exhibit complementary effects in feature modeling.

Regarding the selection of the backbone structure, RepVGG-B1g4 and RepVGG-B1g2 were benchmarked against the RepVGG-D2se architecture adopted in the proposed HPNet. Among the evaluated ablation configurations, RepVGG-D2se combined with SCFE and LSTM consistently achieved the lowest MAE values. Compared with the model using the RepVGG-B1g4 backbone with SCFE and LSTM, the proposed HPNet reduced the yaw, pitch, roll, and Avg MAE values by 2.38°, 3.99°, 3.23°, and 3.2°, respectively. Furthermore, compared with the model using the RepVGG-B1g2 backbone with SCFE and LSTM, the corresponding reductions were 2.7°, 3.73°, 3.29°, and 3.24°, respectively. These results indicate that the RepVGG-D2se backbone provides stronger feature representation capability for rear-view head pose estimation.

### 4.3. Applications of HPNet on CROM Measurement

Since the research/clinical-grade CROM device (the benchmark) outputs only positive values for head movement in any direction (left/right rotation, flexion/extension, and lateral bending) relative to the central zero position, the absolute values of the HPNet angular estimates were used for comparison.

**Figure 6:**
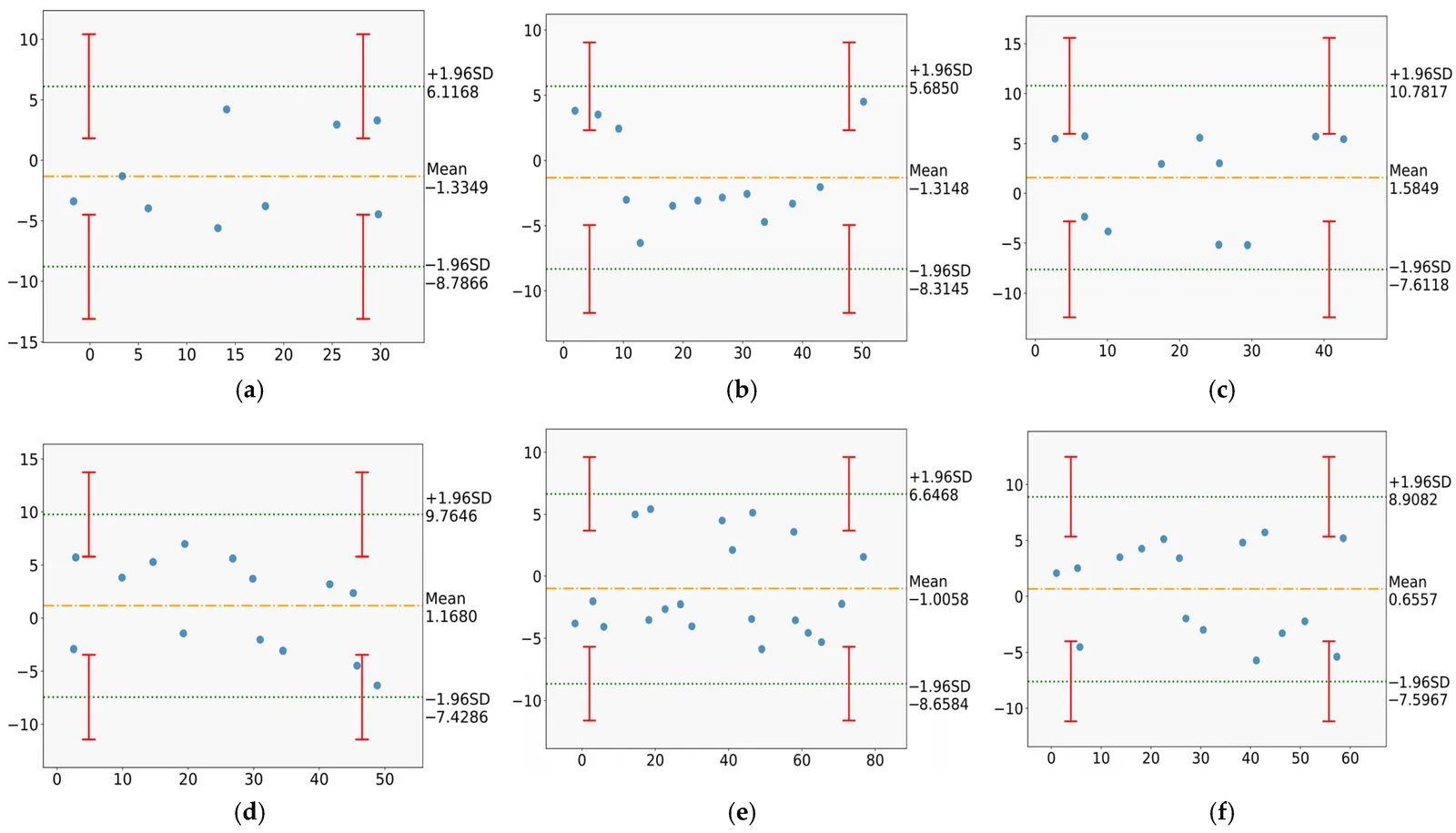
The Bland–Altman plots of the female participant between the CROM device method and the HPNet method for all six cervical movements. The dashed line represents the mean of differences (bias); the dotted lines represent the upper and lower boundaries of 95% limits of agreement (LoAs); and the error bars (solid lines) represent the 95% confidence intervals (CIs) of the boundaries of 95% LoAs. (**a**) Forward Flexion (FF), (**b**) Backward Extension (BE), (**c**) Left Lateral Flexion (LLF), (**d**) Right Lateral Flexion (RLF), (**e**) Left Rotation (LR), and (**f**) Right Rotation (RR).

**Figure 7:**
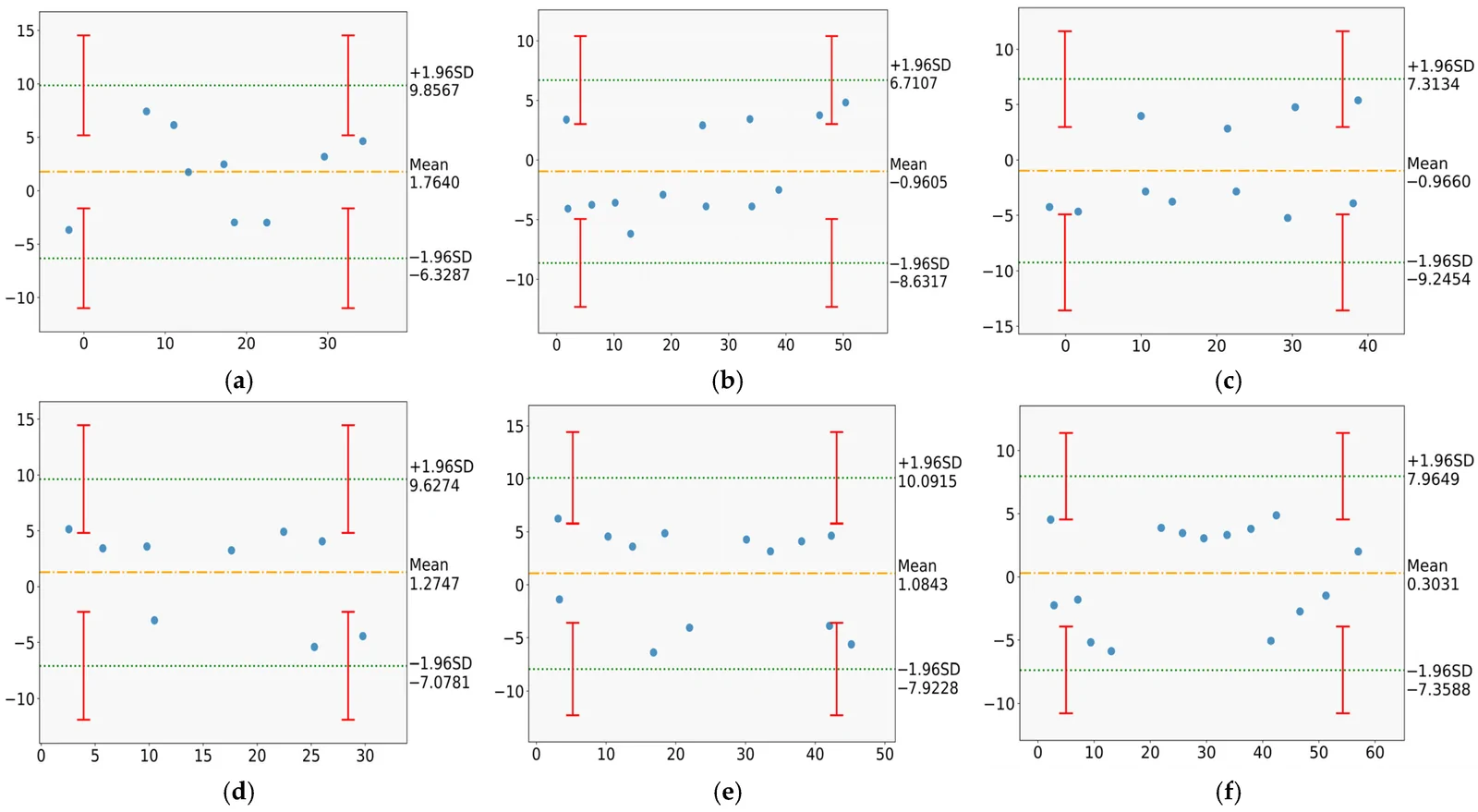
The Bland–Altman plots of the male participant between the CROM device method and the HPNet method for all six cervical movements. The dash dotted line represents the mean of differences; the dotted lines represent the upper and lower boundaries of 95% limits of agreement (LoAs); and the error bars (solid lines) represent the 95% confidence intervals (CIs) of the boundaries of 95% LoAs. (**a**) Forward Flexion (FF), (**b**) Backward Extension (BE), (**c**) Left Lateral Flexion (LLF), (**d**) Right Lateral Flexion (RLF), (**e**) Left Rotation (LR), and (**f**) Right Rotation (RR).

In Experiment 1, Table 4 summarizes the error analysis for one randomly selected male participant and one randomly selected female participant, serving as representative individual-level examples. The Bland–Altman plots (Figure 6 and Figure 7) showed that the mean bias values were close to zero for all six movements in both the female and male participants, indicating no systematic offset between the two methods. The vast majority of data points fell within the 95% LoAs across different movements. A small number of points approached the LoA boundaries, primarily in lateral flexion and rotation movements, which is attributable to reduced discriminative features under large-angle rear-view conditions.

For Experiment 2, the full ranges of cervical angles measured across all participants and trials were: 46.0–88.7° for LR, 40.0–80.0° for RR, 32.7–74.7° for FF, 40.0–96.7° for BE, 29.3–61.3° for LLF, and 27.3–61.3° for RLF. Within these measured ranges, agreement between HPNet and the clinical-grade CROM device was quantitatively evaluated using ICC, MAE, and RMSE (Table 5). The overall results showed an averaged ICC (2,1) of 0.939, an averaged MAE of 4.85°, and an averaged RMSE of 5.89°, indicating excellent consistency between the two measurement methods. ICC (2,1): an intraclass correlation coefficient based on a two-way random-effects model with absolute agreement.

## 5. Discussion

This paper has presented a novel non-contact, privacy-friendly and deep learning-based HPNet model for CROM measurement. The model, which incorporates a RepVGG-D2se backbone, an SCFE block, and an LSTM module, appears to outperform several state-of-the-art algorithms on the CMU Panoptic dataset, achieving MAE values of 3.48°, 3.22°, and 3.34° for yaw, pitch, and roll, respectively. Ablation experiments conducted on the rear-view subset of the CMU Panoptic dataset demonstrated that progressively introducing the SCFE block and the LSTM module consistently improves estimation accuracy.

### 5.1. Reliability of HPNet-Based CROM Measurement

**Figure 8:**
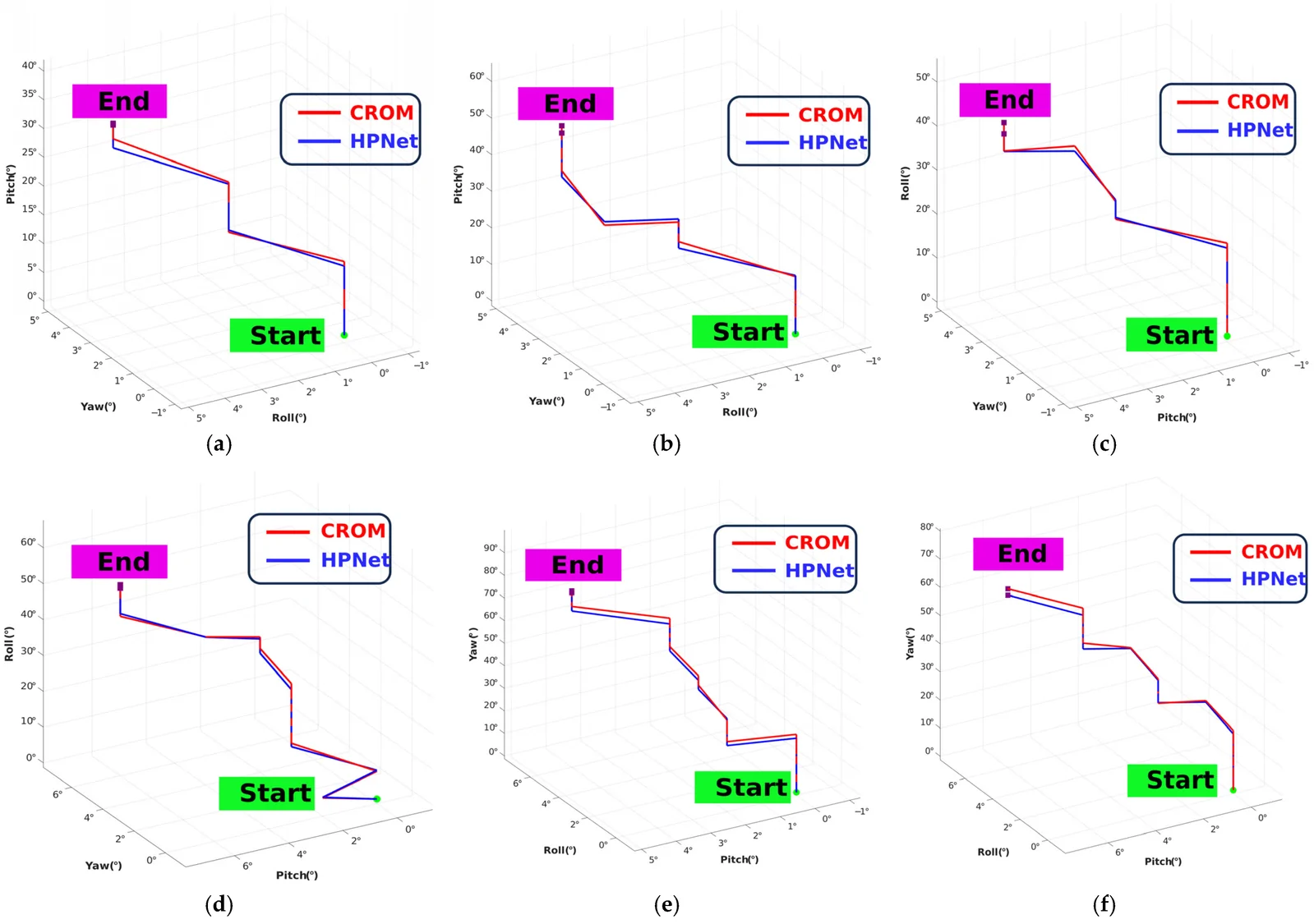
Cervical movement trajectory curves of the female participant (CROM vs. HPNet). (**a**) Forward Flexion (FF), (**b**) Backward Extension (BE), (**c**) Left Lateral Flexion (LLF), (**d**) Right Lateral Flexion (RLF), (**e**) Left Rotation (LR), and (**f**) Right Rotation (RR).

**Figure 9:**
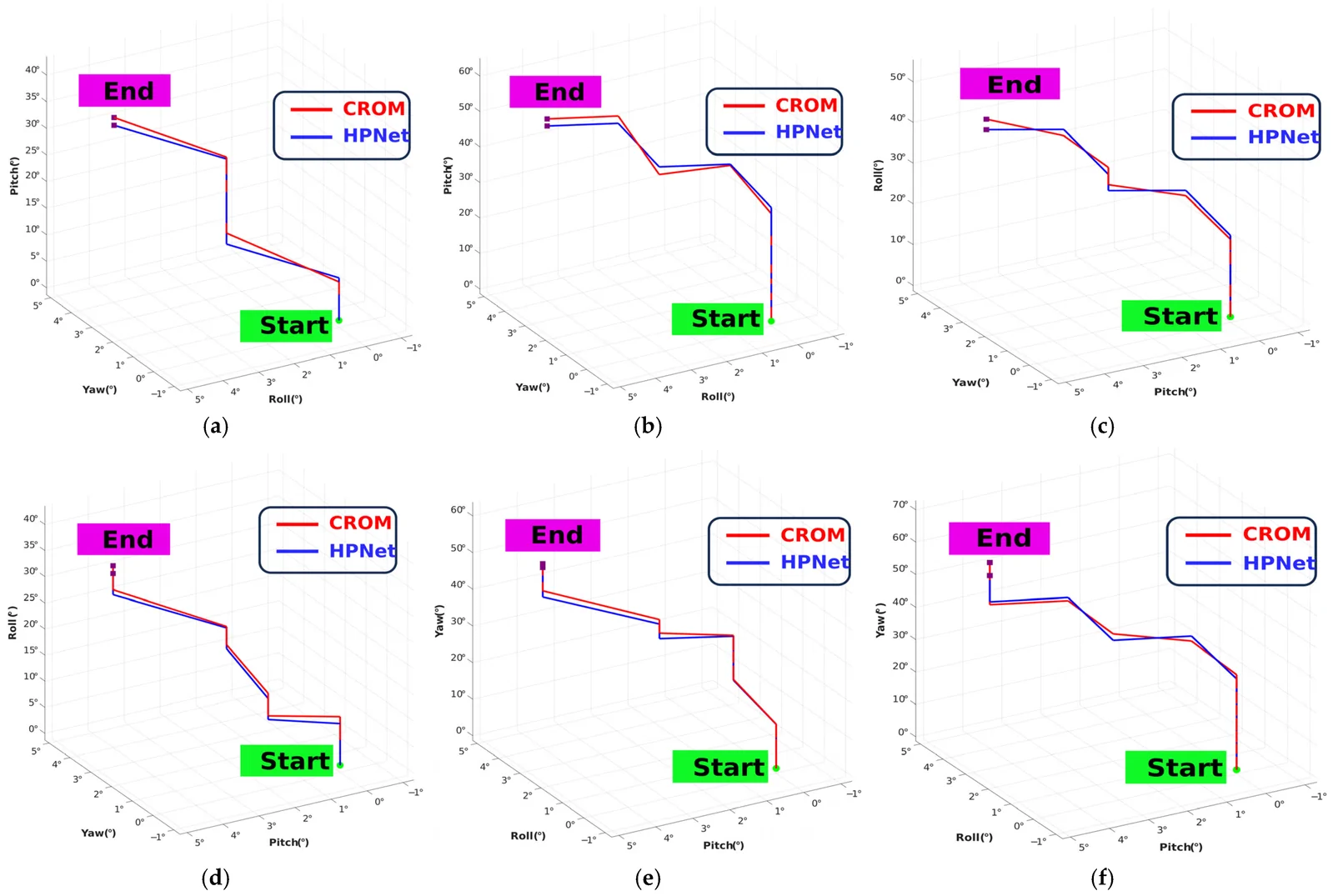
Cervical movement trajectory curves of the male participant (CROM vs. HPNet). (**a**) Forward Flexion (FF), (**b**) Backward Extension (BE), (**c**) Left Lateral Flexion (LLF), (**d**) Right Lateral Flexion (RLF), (**e**) Left Rotation (LR), and (**f**) Right Rotation (RR).

Two experiments were used to validate the feasibility and reliability of HPNet for CROM measurement against a research/clinical-grade reference. ICCs > 0.91 for all six cervical movements confirmed the presence of a satisfactory measurement consistency between the two methods. In addition, HPNet estimated yaw, pitch, and roll in 38.07 ms, faster than manual CROM readings. Although the cervical mark cap (CMC) [46] achieved comparable performance for all six cervical movements, it was tested on only one male subject. Additionally, the CMC system required the participant to wear a customized cap and required three smart phones to track the markers on the cap. In contrast, HPNet provided a completely marker-free solution that needed only a single rear-positioned smartphone and demonstrated high repeatability in both feasibility (one male and one female) and reliability (40 participants) assessments. Moreover, the Bland–Altman plots exhibited that the mean bias values were close to zero for all six movements in both male and female participants, with the vast majority of data points falling within the 95% LoAs. The LoA bandwidth ranged from approximately 14° to 18° across different movements, reflecting acceptable random variability in single measurements. These results surpassed those of both the VR device-based [6] and CMC [46] systems. In Experiment 1, the HPNet-estimated angles for each movement performed by the male and female participants were plotted in 3D pitch–yaw–roll angular space and connected sequentially to form cervical motion trajectories. For comparison, angles measured by the research/clinical-grade CROM device for the same participants were overlaid in the same coordinate system (Figure 8 and Figure 9).

The resulting trajectories, generated by sequentially connecting the HPNet-estimated angles, empirically showed that HPNet was able to closely follow the dynamic profiles of the reference device across the tested range of motion. Since 3D trajectories capture more information than static data, and movement patterns differ between healthy individuals and patients [45], this approach holds promise for both clinical and home-based cervical assessments. Specifically, cervical motion curves could support the full-cycle management of diagnosis, treatment, and follow-up, while also enabling the development of a historical CROM database to help clinicians track the changes in the severity or recovery of conditions that affect cervical range of motion and its control over time.

Compared with IMU-based CROM measurement systems [7], our approach eliminates the need for frequent calibration [7,53] or additional hardware [54,55], significantly reducing the system complexity and cost. Furthermore, the rear-facing camera can effectively preserve privacy and eliminate the need for wearing any cumbersome apparatus. In this study, “marker-free” indicates that no external markers, wearable sensors, customized caps, or head-mounted devices were attached to the participants during all six cervical movement modes. This marker-free approach is especially beneficial for patients with cervical spondylosis [56] or sports-related injuries [43], for whom even minimal contact can exacerbate symptoms.

### 5.2. Comparison with State-of-the-Art HPE Methods

As image-based CROM measurement remains an emerging technique, only a limited number of studies have been reported to date [9,14,15,16]. Among these, the most recent state-of-the-art study was conducted by Ritthipravat et al. [9], who employed a frontal webcam to capture facial RGB images. Their pre-processing pipeline included face detection, facial landmark localization, and loose cropping to a resolution of 224 × 224 pixels. Although such a pipeline is widely adopted in conventional head pose estimation tasks, it captures and exposes facial biometric information, which raises substantial ethical and privacy concerns in clinical and telehealth applications. In contrast, the proposed HPNet system uses a rear-facing smartphone camera placed 60 cm behind participants to image the occipital region, thereby completely eliminating facial data and achieving greater privacy preservation. For network architecture, Ritthipravat et al. [9] utilized EfficientNet-Lite4 as the backbone, integrated with multi-level attention modules and a modified Atrous Spatial Pyramid Pooling (ASPP) module for enhanced feature representation, followed by multi-bin classification for Euler angle regression. By means of comparison, the proposed HPNet adopts RepVGG-D2se as the backbone, coupled with a SCFE module and an LSTM-based feature refinement unit. Furthermore, HPNet employs a 6D rotation representation to avoid gimbal lock, thereby improving estimation stability during large-amplitude neck rotations. In addition, the present study implemented stepwise incremental angle measurements and a more comprehensive statistical validation, including intraclass correlation coefficient (ICC) and Bland–Altman analysis, neither of which was reported in the previous work [9].

Regarding prediction performance, both methods achieve clinically acceptable accuracy for CROM estimation; however, the HPNet system demonstrates superior reliability and greater potential for real-world clinical translation. On public head pose datasets, Ritthipravat et al. [9] reported averaged MAE values of 3.50°, 3.40°, and 2.31° on three benchmark datasets using frontal facial images. For real clinical CROM measurement, their method yielded an averaged MAE of 4.58°. In contrast, the proposed HPNet achieved state-of-the-art performance on the rear-view subset of the CMU Panoptic dataset, with MAE values of 3.48° for yaw, 3.22° for pitch, and 3.34° for roll, corresponding to an overall mean MAE of approximately 3.35°. For in-house CROM measurements, HPNet maintained an averaged MAE value of 4.85°, with all errors falling within clinically acceptable ranges [9]. More importantly, the HPNet system achieved excellent inter-instrument reliability, with ICC values above 0.91 for all movement directions. Bland–Altman plots further confirmed negligible systematic bias and acceptable 95% LoAs, both of which are critical for clinical deployment.

**Figure 10:**
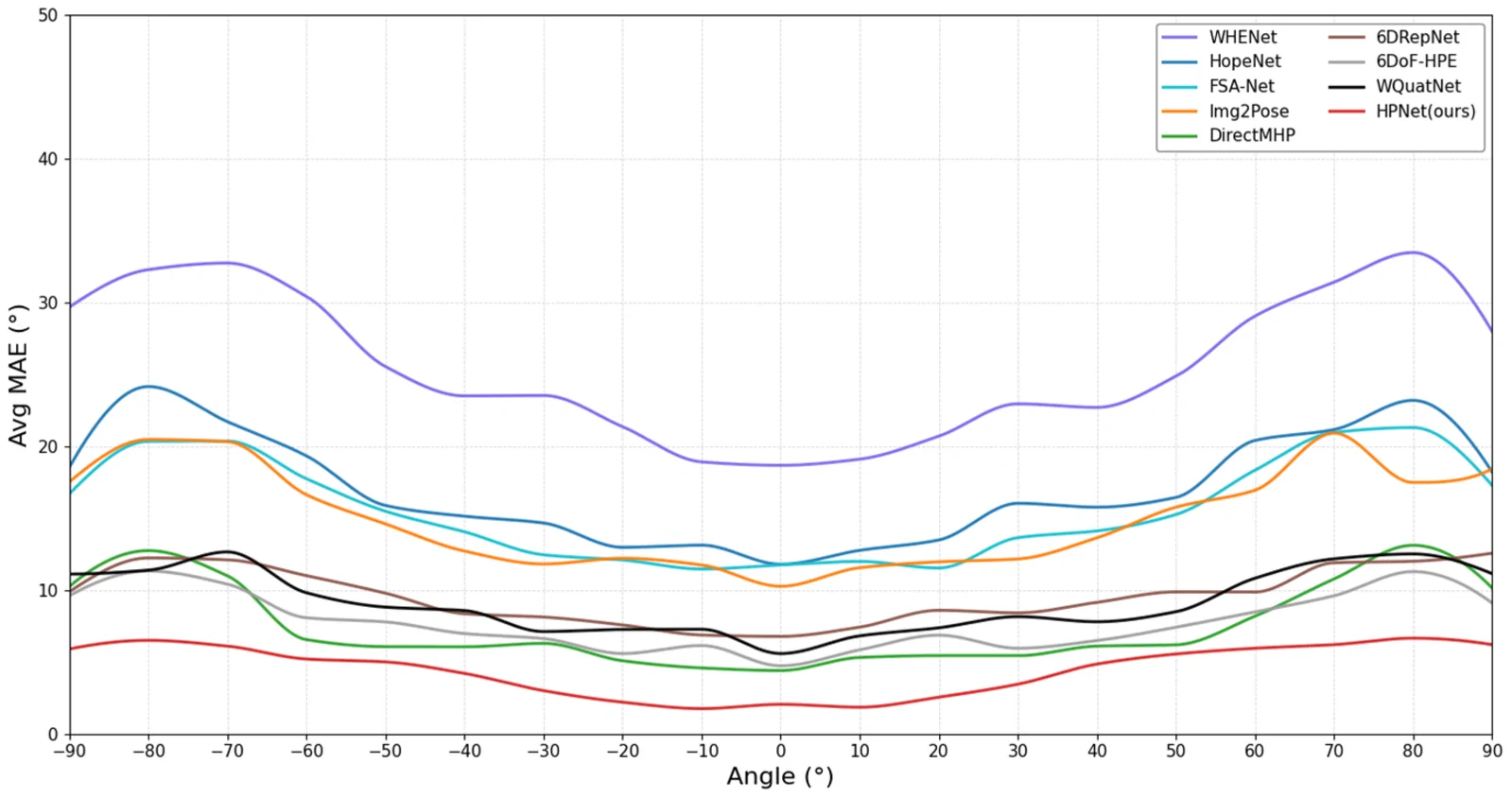
Comparison of different methods across yaw angles ranging from −90° to 90° on the rear-view validation subset of the CMU Panoptic dataset.

To provide a more detailed MAE comparison similar to Ref. [49], we performed an angle-range-based MAE analysis on the rear-view validation subset of the CMU Panoptic dataset. As shown in Figure 10, all HPE methods listed in Table 2 were compared over yaw angles ranging from −90° to 90° [49]. HPNet achieved the lowest averaged MAE across most angle ranges. Although the MAE slightly increased at larger absolute angles, HPNet still maintained a clear advantage over the other compared methods, indicating more stable and robust estimation performance across different head pose ranges.

### 5.3. Limitations

A key limitation of this study is that the reliability validation was performed in a cohort of healthy young adults aged 18–27 years. However, this population provides a reasonable starting point for evaluating the feasibility and agreement of a CROM measurement system [49], since the basic anatomical structures and kinematic principles underlying cervical range-of-motion assessment are shared by healthy individuals and patients with cervical disorders. Therefore, the present findings demonstrate the feasibility and reliability of HPNet in standard CROM measurement tasks and provide a basis for future validation in broader age groups and clinical patient populations. Future work must recruit participants across a wider age range and with diverse pathologies, in order to show usefulness in a clinical situation. Furthermore, although photographs were acquired immediately after CROM recording in Experiment 1, removing the device required small but unavoidable head movements, resulting in a mean postural drift of 1.2° (SD = 0.6°) between the CROM and photographic measurements, which was below the 2° measurement resolution of the CROM device [57]. This protocol was chosen to show that HPNet could operate without any markers. It is worth noting that the MAE in Experiment 2 (40 participants, MAE 4.33–5.22°) was slightly higher than that in Experiment 1 (2 participants, MAE 3.50–4.58°), while the ICC decreased from above 0.926 to above 0.913. The larger cohort introduces greater inter-individual variability in anatomy, head shape, and hair characteristics, which would be expected to increase the difficulty of rear-view pose estimation. In the current implementation, rear-view images were first captured using the smartphone camera, and YOLO26 was then used as the front-end localization module to detect the occipital region and provide region-of-interest inputs for HPNet. However, for broader practical applications where head localization is required—such as in scenarios with varying distances—YOLO26, which was validated in our head detection experiments with a mAP@50 of 0.984, can be employed as the front-end detection module [39]. This approach would enable real-time head region localization even under diverse postures and scale variations, providing stable region-of-interest inputs for subsequent pose estimation. Although this study was to act as a proof of concept, the apparent success in terms of outcome supports further development. In such future work, we will conduct systematic on-device deployment experiments on representative mid-range smartphones, including end-to-end latency, FPS, device-level power consumption, and optimization strategies such as quantization, pruning, and hardware acceleration. It should be noted that HPNet has thus far been implemented and validated primarily on a computer-based platform and has not yet been deployed on smartphones. Therefore, its real-time inference performance and practical feasibility on mobile devices remain to be systematically evaluated in future work. Future work will investigate deployment strategies for real-world applications, including mobile security, data protection, and standardized clinical workflows, to enhance the practical applicability of the proposed method. To prevent unauthorized access to the phones, data encryption, secure storage, identity protection and application permission management are issues to be addressed later during practical system deployment.

## 6. Conclusions

In this work, privacy preservation specifically refers to avoiding the exposure of subjects’ frontal/facial information. Unlike methods relying on frontal face images for head pose estimation, our model only adopts rear-view images of the human body, so that facial identity features will not be captured and the risk of facial privacy leakage can be mitigated. This paper introduces HPNet, a novel architecture for automated, non-invasive and privacy-protected CROM assessment. At its core, RepVGG-D2se serves as the backbone network, augmented by the SCFE module to enhance spatial awareness and pose-related feature representation. The LSTM module served as a gated feature refinement unit, providing adaptive feature selection and implicit regularization for the nonlinear mapping from high-dimensional convolutional features to 6D rotation parameters. On the public CMU Panoptic dataset, HPNet achieved the lowest MAE values of 3.48° for yaw, 3.22° for pitch, and 3.34° for roll when compared with state-of-the-art algorithms.

The comparative experiments between HPNet and the research/clinical-grade CROM device reflected a high level of agreement between the two techniques, while HPNet works faster than manually reading data from the clinical device. In conclusion, the proposed HPNet appears to be capable of accurately obtaining 3D CROM information with the advantages of automation, non-invasiveness, and privacy protection without the need for assistance from clinically trained professionals. Although the use of a smartphone camera to obtain useable images indicates that the technology is present to support the development of a measurement system on a mobile device, the computing power and size of the program presently make this unlikely without further development.

## Figures and Tables

**Table 1:** Demographic information of all participants reported as mean ± 1 standard deviation.

Participants	Age (Years)	Height (cm)	Body Mass (kg)
Male (20)	23.2 ± 1.6	176.7 ± 4.8	70.4 ± 8.1
Female (20)	22.3 ± 1.4	164.6 ± 1.8	53.1 ± 5.2
Overall (40)	22.7 ± 1.6	170.7 ± 7.1	61.8 ± 11.1

**Table 2:** Performance comparison and parameter analysis of different HPE methods.

Method	Retrained	Params. (M)	MAE			
			Yaw (°)	Pitch (°)	Roll (°)	Avg MAE (°)
WHENet [44]	No	4.4	37.96	22.7	16.54	25.73
HopeNet [48]	No	23.9	20.40	17.47	13.40	17.09
FSA-Net [32]	No	1.2	17.52	16.31	13.02	15.62
Img2Pose [31]	No	42.3	12.99	16.69	15.64	15.11
DirectMHP [51]	Yes	12.4	6.29	8.98	7.41	7.56
6DRepNet [33]	Yes	39.3	8.38	11.24	9.19	9.60
6DoF-HPE [39]	Yes	34.1	6.72	8.90	7.77	7.80
WQuatNet [52]	Yes	117.8	8.5	9.97	9.12	9.20
HPNet (ours)	Yes	139.3	3.48	3.22	3.34	3.35

**Table 3:** Performance comparison of different backbone architectures and module combinations in the ablation study using the CMU Panoptic dataset.

Experiment Setting	Model Configuration/Test Setting	MAE			
		Yaw (°)	Pitch (°)	Roll (°)	Avg (°)
Ablation trial	RepVGG-B1g4	6.38	7.65	7.28	7.10
Ablation trial	RepVGG-B1g4 + SCFE	6.25	7.56	7.22	7.01
Ablation trial	RepVGG-B1g4 + LSTM	6.19	7.58	7.17	6.98
Ablation trial	RepVGG-B1g4 + SCFE + LSTM	5.86	7.21	6.57	6.55
Ablation trial	RepVGG-B1g2	6.73	7.49	7.21	7.14
Ablation trial	RepVGG-B1g2 + SCFE	6.56	7.38	7.12	7.02
Ablation trial	RepVGG-B1g2 + LSTM	6.51	7.32	7.05	6.96
Ablation trial	RepVGG-B1g2 + SCFE + LSTM	6.18	6.95	6.63	6.59
Ablation trial	RepVGG-D2se	5.58	6.85	6.52	6.32
Ablation trial	RepVGG-D2se + SCFE	5.42	6.72	6.38	6.17
Ablation trial	RepVGG-D2se + LSTM	5.37	6.68	6.31	6.12
Ablation trial	RepVGG-D2se + SCFE + LSTM	3.48	3.22	3.34	3.35

**Table 4:** Comparison of variability and error metrics for all six cervical movements for the randomly selected female and male participants.

Movement	Male			Female		
	ICC (2,1)	MAE (°)	RMSE (°)	ICC (2,1)	MAE (°)	RMSE (°)
FF	0.952	3.90	4.27	0.935	3.66	3.82
BE	0.973	3.77	3.88	0.978	3.50	3.67
LLF	0.940	4.04	4.14	0.960	4.58	4.74
RLF	0.968	4.13	4.21	0.926	4.06	4.38
LR	0.986	4.36	4.54	0.956	3.73	3.93
RR	0.975	3.55	3.78	0.978	3.92	4.12

**Table 5:** Comparison of the measurement variability and error metrics for all six cervical movements across all participants.

Movement	Male (20)			Female (20)		
	ICC(2,1)	MAE (°)	RMSE (°)	ICC(2,1)	MAE (°)	RMSE (°)
FF	0.949	5.05	6.43	0.945	4.33	5.06
BE	0.965	4.85	6.33	0.938	4.43	5.28
LLF	0.941	5.17	5.71	0.913	5.10	6.13
RLF	0.928	5.20	6.29	0.945	4.39	5.44
LR	0.944	4.53	5.51	0.936	4.92	6.12
RR	0.932	4.98	6.22	0.934	5.22	6.21

## Data Availability

The data presented in this study are available on request from the corresponding author.
